# F148. A PILOT STUDY OF [11C] (R)-MEQAA PET BRAIN IMAGING ANALYSIS OF ALPHA 7 NICOTINIC ACETYLCHOLINE RECEPTORS AVAILABILITY IN SCHIZOPHRENIA

**DOI:** 10.1093/schbul/sby017.679

**Published:** 2018-04-01

**Authors:** Tomoyasu Wakuda, Masamichi Yokokura, Kyoko Nakaizumi, Yasuhiko Kato, Yosuke Kameno, Masami Futatsubashi, Etsuji Yoshikawa, Yasuhiro Magata, Yasuomi Ouchi, Hidenori Yamasue, Nori Takei

**Affiliations:** 1 Hamamatsu University School of Medicine; 2 Hamamatsu Photonics KK; 3 Hamamatsu University School of Medicine, Institute of Psychiatry

## Abstract

**Background:**

A growing body of evidence suggests that the aberrant cholinergic system may underlie the pathophysiology in schizophrenia. Nicotinic acetylcholine receptor (nAChR) subtype α7 (henceforth ‘α7 nAChR’) is located in presynaptic and postsynaptic constructs in the cerebral cortex and considered to play a key role in the regulation of learning and memory. Additionally, α7 nAChR is deemed to exert neuroprotective effects. Therefore, α7 nAChR is one of the potent therapeutic targets for negative symptoms and cognitive impairment in schizophrenia. In effect, several randomised trials to assess the efficacy and safety of α7 nAChR agonists are currently underway.

There is some evidence in support of aberrant α7 nAChR in schizophrenia. In postmortem studies, protein levels of α7 nAChR in the frontal cortex (Guan et al., 1999) have been reported to be decreased in patients with schizophrenia. However, the availability of α7 nAChR in individuals with schizophrenia has yet to be examined in vivo. In this pilot study, we aim to clarify availability of α7 nAChR in the brains of patients with schizophrenia using positron emission tomography (PET) with a ligand of [11C](R)-2-methylamino-benzoic acid 1-aza-bicyclo[2.2.2]oct-3-yl ester ([11C](R)-MeQAA).

**Methods:**

All participants provided informed consent. Inclusion criteria included diagnosis of schizophrenia according to the Diagnostic and Statistical Manual of Mental Disorders-Fifth Edition (DSM-5, 2013). Patients were excluded if they had (1) full IQ under 69 measured with the Wechsler Adult Intelligent Scale-III; (2) current or past history of tobacco smoking; (3) history of neurological disorder or structural brain abnormality; (4) use of benzodiazepines, antidepressants, or anticholinergics in the past 6 months; and (5) substance abuse. Although scanning drug-free or drug-naïve patients for investigation is optimal, it is extremely difficult to attain this. Consequently, participants with schizophrenia comprised medicated cases.

We evaluated the availability of α7 nAChR by estimating non-displaceable binding potential (BPND) of the tracer using PET with [11C] (R)-MeQAA, a selective PET tracer for α7 nAChR. Four patients with schizophrenia (age: range 27–39; m/f: 2/2) and 5 age-matched healthy adults (age: range 22–32; m/f: 2/3) underwent the PET scan. The level of BPND in patients with schizophrenia was compared with that for control participants by applying regions of interest (ROIs) approach. In this pilot study, we opted for 4 cortical areas, the superior frontal, middle frontal, parietal, and temporal cortices, for ROIs. This study was approved by the Hamamatsu University School of Medicine Ethics Committee.

**Results:**

We found the levels of [11C] (R)-MeQAA BPND significantly lower in the middle frontal cortex (p = 0.036) in patients with schizophrenia. Additionally, there was a trend towards a decreased level of BPND in the temporal cortex (p = 0.067) and parietal cortex (p = 0.087) in the brains of schizophrenia patients, although it failed to reach statistical significance. There was no difference in the superior frontal cortex.

**Discussion:**

To our knowledge, this represents the first demonstration of anomalies in the acetylcholinergic system in the in vivo brains of schizophrenia patients. However, this is regarded as a pilot study, and further recruitment of schizophrenia patients with a recent onset and minimal use of antipsychotic medication, followed by scanning and data analyses, will be continued.

